# Discrepancy between the effects of morronside on apoptosis in human embryonic lung fibroblast cells and lung cancer A549 cells

**DOI:** 10.3892/ol.2014.1850

**Published:** 2014-02-03

**Authors:** JIAN-PING CHEN, DE-GUO XU, XIAO-YI YU, FENG-MING ZHAO, DONG-QING XU, XU ZHANG, BAO-CHANG CAI, MING-YAN WANG

**Affiliations:** 1 Jiangsu Key Laboratory of Chinese Medicine Processing, Nanjing University of Chinese Medicine, Nanjing, Jiangsu 210029, P.R. China; 2 China Pharmaceutical University, Nanjing, Jiangsu 210009, P.R. China

**Keywords:** human embryonic lung fibroblasts cells, apoptosis, morroniside, retinoblastoma protein

## Abstract

Morroniside is a water-soluble compound extracted from the fruit of *Cornus officinalis* and is used to protect lung activity against aging. In the present study, the manner in which morroniside regulates normal lung and cancer cells was examined. The human embryonic lung fibroblast (HELF) cell line and lung cancer A549 cell line, and their responses to morroniside treatment, were examined. Results showed that morroniside reverses the apoptotic effect of H_2_O_2_ on HELF cell growth, protecting cell proliferation and normal cell morphology and inhibiting apoptosis. However, these effects were not present in A549 cells. Western blotting showed that morroniside also markedly downregulated retinoblastoma protein in HELF cells. These results suggest that morroniside treatment exhibits different effects on apoptosis in HELF and A549 cells, making it a viable compound for decreasing the side effects of anticancer medicines in normal cells.

## Introduction

The fruit of *Cornus officinalis* is effective for treating liver and lung disease in traditional Chinese medicine (1). In recent decades, the pharmacology and phytochemistry of the fruit of *Cornus officinalis* have been extensively investigated. Clinical studies have demonstrated that this fruit has anticancer and antioxidative roles (2,3). Based on various clinical roles, several effective water-soluble components of the fruit of *Cornus officinalis* have been identified. For instance, gallic acid has antitumor properties (4) and oleanolic acid has antiulcer properties and markedly reduces blood pressure and blood glucose levels (5). Morroniside is used to suppress the generation of superoxide anions in a concentration-dependent manner, and protects nerve cells from H_2_O_2_-induced oxidation injury (6,7). At the molecular level, morroniside can enhance the activity of alkaline phosphatase and decrease caspase-3 mRNA levels (8). The protection of cell growth by morroniside is hypothesized to be involved in regulation of cell proliferation and apoptosis.

The retinoblastoma (Rb)/pocket protein family is made up of tumor suppressor proteins involved in cell proliferation, apoptosis and cell differentiation (9,10). The Rb protein is one of the master regulators of the eukaryotic cell cycle, regulating the G1/G0 phase and also the S and G2 phases (11). Rb protein activity is regulated by phosphorylation. In normal cells during the M-to-G1 transition, the Rb protein is progressively dephosphorylated by protein phosphatase 1, enabling it to bind E2F, blocking transcription of multiple genes involved in cell cycle progression and elongating the G1/G0 phases. Following mitogenic stimulation of the cell, the Rb protein is phosphorylated by cyclin-dependent kinase (CDK) 4/6-cyclin D complexes. Phosphorylation inactivates the Rb protein, resulting in release of E2F, enabling E2F to transcriptionally activate genes that facilitate the G1/S transition and S phase. The Rb protein remains phosphorylated/inactive throughout the S, G2 and M phases through hyperphosphorylation by cyclin E/CDK2 complexes (12–15).

The present study showed that morroniside decreases cell apoptosis induced by H_2_O_2_ in HELF cells but not in A549 cells. Additionally, the S phase of the cell cycle was returned to normal function and Rb protein levels were downregulated by morroniside. These results indicate that the effects of morroniside on HELF and A549 cells may correlate with the regulation of Rb protein levels and S phase length.

## Materials and methods

### Cells and reagents

HELF and A549 cells were purchased from Shanghai Institute of Biochemistry and Cell Biology (Shanghai, China). Morroniside was provided by Jiangsu ZhongKang New Drug Fingerprint R&D Co., Ltd (Nanjing, China) at a purity of >98%; MTT, acridine orange (AO), ethidium bromide (EB), RNase A, propidium iodide (PI) and trypsin were purchased from Sigma (St. Louis, MO, USA). RPMI 1640 medium was obtained from Gibco-BRL (Carlsbad, CA, USA) and fetal bovine serum (FBS) was from Sijiqing Biotechnology (Hangzhou, China). Cell culture and drug incorporation. HELF and A549 cells were cultured in RPMI 1640 medium with 10% (v/v) FBS, 100 U/ml streptomycin and 100 U/ml penicillin, at 37°C with 5% CO_2_. The cells digested with 0.25% trypsin were cultured in 96-well plates (for proliferation analysis), 6-well plates (for morphology observation) or flasks (for cell cycle analysis). In all experiments, HELF/A549 cells were cultured without treatment (normal control), with 300 μM H_2_O_2_/0.2 nM staurosporine (model) or with combined treatment of H_2_O_2_/staurosporine and morroniside at various doses (12.5, 25, 50, 100 and 200 μg/ml).

### Cell proliferation analysis

Cell proliferation was determined by MTT assay. HELF cells in exponential growth were seeded at a final concentration of 6×10^4^ cells/ml in a 96-well plate. At 50% confluency, the cells were pretreated with morroniside at various concentrations for 2 h, exposed to H_2_O_2_ or staurosporine (0.2 nM) and incubated for 48 h. Next, 50 ml MTT (1 mg/ml) was added to each well, followed by incubation for 4 h at 37°C. The supernatants were discarded and the formazan product was dissolved in 100 μl dimethyl sulfoxide. The 96-well plate was agitated on a micro-vibrator for an additional 10 min. The optical density of each well was measured at λ490 nm using an enzyme-immunoassay instrument.

### Morphological examination

Cell groups were subjected to various designated treatments, as discussed. The cells were observed under a fluorescence microscope and photographed. Cells were seeded at a concentration of 1×10^5^/well in a 6-well culture plate. A cover slip was placed on the bottom of each well to allow cells to grow on the surface as a monolayer. At 40–60% confluency, the cells were divided into three groups: Normal control group; H_2_O_2_-treated group, treated with 300 μM H_2_O_2_ and incubated for 48 h; morroniside plus H_2_O_2_-treated group, pretreated with 100 μg/ml morroniside for 2 h and exposed to 300 μM H_2_O_2_. After a 48-h incubation period, the cell culture was washed with phosphate-buffered saline (PBS), stained with 10 μg/ml AO/EB for 2 min and observed under a fluorescence microscope (Olympus BX60; Olympus, Tokyo, Japan).

### Flow cytometry

Cells were treated as discussed. After 24-h culture, cells were washed twice with ice-cold PBS buffer (pH 7.4) prior to fixing with 75% ice-cold ethanol and staining with PI (1 mg/ml) in the presence of 1% RNAase A for 30 min. The samples were subsequently analyzed by flow cytometry (BD Biosciences, Franklin Lakes, NJ, USA). The Cell Quest software (BD Biosciences) was applied to analyze apoptosis rates and the cell cycle.

### Western blotting

HELF cells were lysed in cold lysis buffer [40 mM HEPES (pH 7.5), 150 mM NaCl, 1.5 mM MgCl_2_, 1 mM EDTA, 1 mM dithiothreitol and 1 mM fresh phenylmethylsulfonyl fluoride]. Lysates were centrifuged at 12,000 × g for 15 min. The supernatant was collected and the protein concentration was determined by Lowry protein assay. Next, 20 μg protein was loaded in each well, electrophoresed by SDS-PAGE and transferred onto nitrocellulose membranes. The membranes were blocked with 5% non-fat dried milk in PBS with 0.5 ml/l Tween-20 for 2 h at room temperature, and incubated overnight at 4°C with primary antibodies against human P16, P27, P53 or Rb proteins, purchased from Beyotime (Nanjing, China). This was followed by incubation with a horseradish peroxidase-conjugated secondary antibody (Bioworld Technology, St. Louis Park, MN, USA) at room temperature for 1 h. Enhanced chemiluminescence was used to detect the results.

### Statistical analysis

The statistical analysis among various groups was performed using one-way analysis of variance with Scheffé’s test. All data were processed with SPSS software (SPSS, Inc., Chicago, IL, USA). The results were expressed as the mean ± SD. P<0.05 and P<0.01 were considered to indicate a statistically significant and highly statistically significant difference, respectively.

## Results

### Morroniside reverses inhibition of cell growth in human embryonic lung fibroblasts cells induced by H_2_O_2_

**Figure 1:**
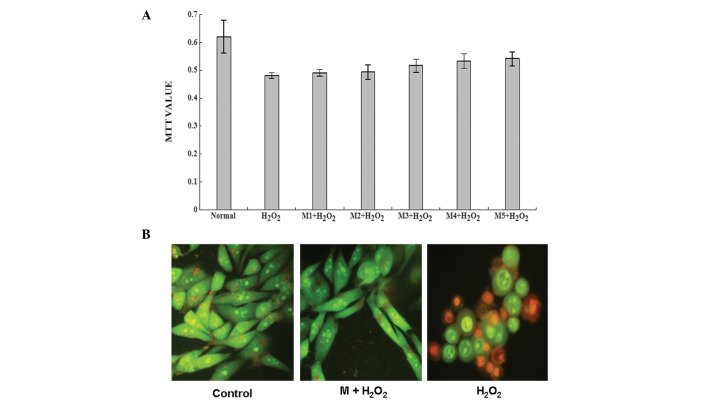
Morroniside reverses H_2_O_2_-induced inhibition of HELF growth and improves cell morphology by preventing cell deformation and maintaining the same appearance as the control group. HELF cells were treated without or with H_2_O_2_ or with a combination of H_2_O_2_ and morroniside at various concentrations. (A) HELF cell growth was measured by MTT. (B) Morphology of HELF cells with acridine orange/ethidium bromide staining. M, morroniside; M1-5, 12.5, 25, 50, 100 and 200 μg/ml morroniside, respectively; HELF, human embryonic lung fibroblast.

Preliminary data indicated that the crude extract of morroniside protected the lung in aging and immune-defective mice. In order to understand whether morroniside regulates the proliferation of lung cells, the growth of HELF cells treated with morroniside (98% purity) was examined. MTT was applied to analyze HELF growth under H_2_O_2_ treatment and with the addition of morroniside at various concentrations (Fig. 1A). Results showed that 300 μM H_2_O_2_ treatment decreased HELF growth, as expected, while 100 μg/ml morroniside markedly increased HELF cell growth and decreased the negative effect on growth under H_2_O_2_ treatment. This indicates that morroniside protects HELF proliferation against H_2_O_2_ treatment.

Additionally, under a fluorescence microscope, staining with mixed dye (AO/EB) showed that, compared with the normal control group, the amount of cells was lower in the model group but increased with the treatment of 100 μg/ml morroniside. The control group was morphologically normal, with evenly-stained bright green nuclei. Under H_2_O_2_ treatment, however, the cells were wrinkled and chromatin was condensed and orange in color. The addition of morroniside morphologically restored the cells back to the morphological appearance of the control group (Fig. 1B). These results indicate that morroniside blocks the negative effect of H_2_O_2_ treatment on HELF cells.

### Morroniside specifically antagonizes H_2_O_2_-induced HELF apoptosis

**Figure 2:**
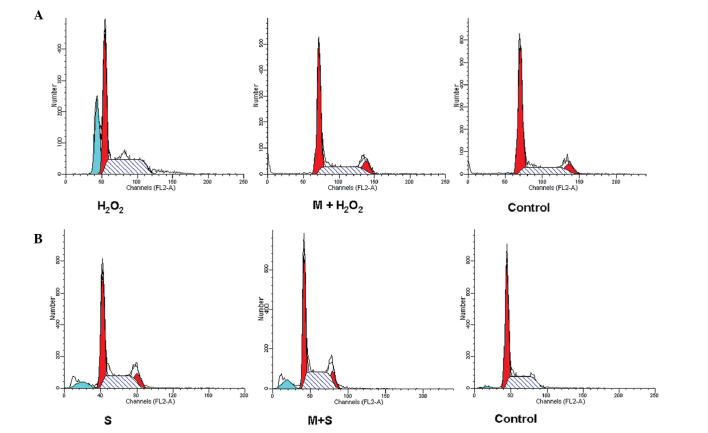
Morroniside antagonizes H_2_O_2_-induced apoptosis in HELF cells but not in A549 cells. (A) Flow cytometry of HELF apoptosis. In 300 μM H_2_O_2_-treated HELF cells, the apoptotic rate was 28.3% following 24-h culture, while the addition of 100 μg/ml morroniside 2 h prior to H_2_O_2_ treatment inhibited HELF cell apoptosis. (B) Morroniside did not inhibit the apoptosis of HELF cells induced by staurosporine. Following 0.2 nM staurosporine treatment of HELF cells, the apoptotic rate was 8.1% after 24-h treatment. However, the addition of 100 μM morroniside 2 h prior to staurosporine treatment showed a similar apoptotic rate of 8.3%. M, morronside; S, staurosporine; HELF, human embryonic lung fibroblast. Blue, apoptotic cells; red, normal cells.

H_2_O_2_ treatment can result in various consequences in terms of cell growth, including apoptosis, autophagy and necrosis (16). To understand whether morroniside treatment is involved in the regulation of apoptosis, flow cytometry was used to analyze the cell cycle and rate of apoptosis in HELF cells under various treatments (Fig. 2A). Results demonstrated that 300 μM H_2_O_2_ induced a 41% rate of apoptosis in HELF cells (control group apoptosis rate, 0.48%), however, the addition of morroniside (100 μg/ml) 2 h prior to H_2_O_2_ treatment maintained the apoptotic rate at the normal control level, indicating that morroniside protected HELF cells from the H_2_O_2_-induced apoptosis. Furthermore, H_2_O_2_ treatment elongated the S phase of the cell cycle, while morroniside markedly decreased the duration of the S phase to that of normal cells, suggesting that morroniside repairs the dysfunctional regulation of the S phase induced by H_2_O_2_. To understand whether morroniside regulates the common signaling pathways of apoptosis, staurosporine was used as an apoptotic model to examine the consequences of morroniside treatment on staurosporine-induced apoptosis (Fig. 2B). Flow cytometry results showed that 0.2 nM staurosporine caused an 8.1% apoptotic rate in HELF cells and the combination of morroniside and staurosporine resulted in a similar apoptotic rate of 8.3%. This suggests that morroniside may not reverse apoptosis induced by staurosporine. Therefore, morronoside is involved in H_2_O_2_ signaling and likely to regulate the upstream apoptotic signaling pathways.

### Morroniside does not reverse apoptosis of lung cancer A549 cells

**Figure 3:**
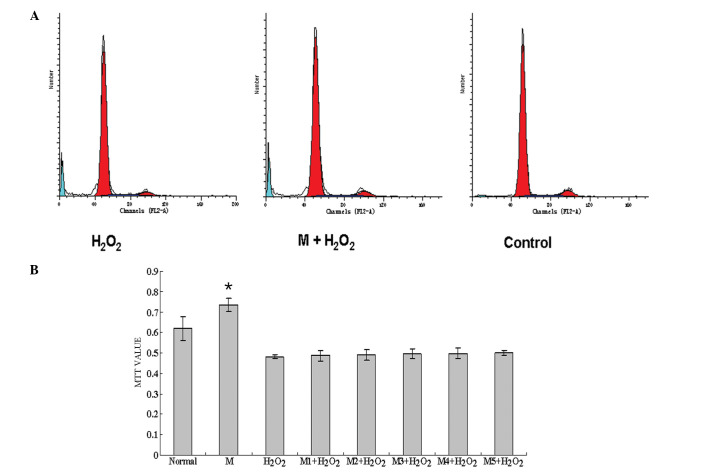
Morroniside does not inhibit the apoptosis of A549 cells induced by H_2_O_2_. (A) A549 apoptosis under various treatments. Treatment with 300 μM H_2_O_2_ induced an 8.1% rate of apoptosis in A549 cells. The addition of 100 μg/ml morroniside 2 h prior to H_2_O_2_ treatment showed a similar apoptotic rate of 7.8%. (B) A549 cell growth was measured by MTT. Compared with the control group, morroniside significantly increased A549 growth, H_2_O_2_ treatment decreased cell growth and combined treatment of morroniside and H_2_O_2_ did not improve cell growth (P<0.01). M, morroniside; M1-5, 12.5, 25, 50, 100 and 200 μg/ml morroniside, respectively. *Compared with the H_2_O_2_ group the addition of morroniside showed no significant difference to cell growth. Blue, apoptotic cells; red, normal cells.

As morroniside restored HELF apoptosis to normal levels, the apoptotic effects of morroniside were examined in cancer cells. The A549 cell line was used for the same experiments and 100 μg/ml morroniside was applied 2 h prior to H_2_O_2_ treatment. Flow cytometry showed that 100 μM H_2_O_2_ induced HELF apoptosis at rate of 8.1%. Notably, the combination of morroniside and H_2_O_2_ caused a 7.8% rate of apoptosis (Fig. 3A) and MTT results showed that morroniside markedly increased A549 proliferation but did not reduce the H_2_O_2_-induced inhibition of A549 cell growth. These primary results indicate that morroniside has no apparent effect on A549 growth under H_2_O_2_ treatment (Fig. 3B).

### Morroniside regulates the S phase of the cell cycle and negatively regulates Rb protein levels

**Figure 4:**
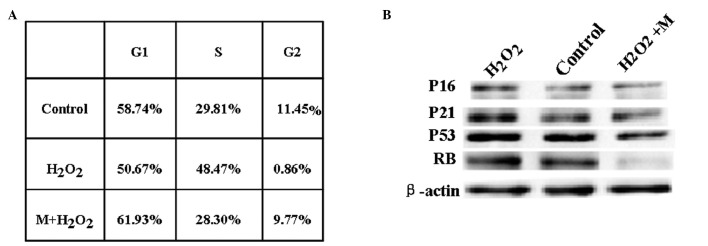
Morroniside mediates S phase and downregulates Rb protein levels. (A) HELF cell cycle under various treatments. The percentage of cells in S phase in the control group was 48.47%. However, the addition of morroniside reduced this to normal levels of ~28.3%, indicating that morroniside regulates S phase in HELF cells. (B) Rb protein levels by western blotting. Results showed that 300 μM H_2_O_2_ treatment did not change Rb protein levels, while the combined treatment of 100 μg/ml morroniside and H_2_O_2_ markedly downregulated Rb protein levels. M, morroniside; HELF, human embryonic lung fibroblast; Rb, retinoblastoma.

In normal HELF cells, flow cytometry showed that the proportion of the cell cycle in the S phase was 28.8% and that H_2_O_2_ increased this to 48.5%. By contrast, morroniside reduced this to 28.3%, indicating that morroniside regulates the cell cycle during the S phase (Fig. 4A). To elucidate the potential signaling pathways regulated by morroniside, the levels of P16, P27, P53 and Rb proteins, all critical factors in the S phase and the regulation of cell growth and apoptosis, were examined (17). Western blotting showed that Rb and P53 proteins were markedly reduced under the combined treatment of morroniside and H_2_O_2_. However, H_2_O_2_ treatment slightly increased P53 protein levels but did not affect Rb protein levels, suggesting that Rb protein levels are not regulated by H_2_O_2_ but may mediate H_2_O_2_-induced apoptosis (Fig. 4B). The loss of Rb protein positively correlated with cell proliferation. These results suggest that the downregulation of Rb protein expression by morroniside may be relevant to the mediation of S phase length.

## Discussion

Previous preliminary experiments (data not shown) have shown that morroniside plays a specific role in the protection of lung cell proliferation, suggesting a potential medicinal use of morroniside to protect normal cells from the side effects of anticancer medication. The present study showed that morroniside exhibits differential effects on apoptosis in HELF and A549 cells under H_2_O_2_ treatment. Notably, morroniside significantly reversed the negative effects of H_2_O_2_ on HELF cell growth but not on A549 cells, indicating that morroniside-regulated signaling pathways are relevant for partially differentiating between the signaling pathways in HELF and A549 cells. Furthermore, morroniside protected the S phase and downregulated Rb protein, suggesting that the molecular mechanisms of morroniside mediating the cell cycle (S phase) may be relevant to Rb protein signaling.

Results of the present study suggest that the different signaling pathways of HELF and A549 cell lines interact with H_2_O_2_ signaling and are specific to the regulation of S phase. H_2_O_2_ treatment was applied to establish a model of hepatocellular oxidative stress, as H_2_O_2_ acts as a harmful factor in these cells, resulting in accumulation of reactive oxygen species (ROS) and an imbalance between the cell oxidation and antioxidant system (18). Results showed that H_2_O_2_ did not increase Rb protein levels, suggesting that morroniside and H_2_O_2_ signaling may interact downstream of the Rb protein. The interaction of signaling pathways regulated by ROS and morroniside requires further investigation. Notably, results also showed that morroniside did not reverse staurosporine-induced apoptosis, indicating that morroniside may not directly target the apoptosis signaling pathways, but acts on the upstream molecules beyond the apoptotic signaling pathways. Furthermore, staurosporine, an inhibitor of protein kinase C, was used to establish an apoptosis model through elongation of G2 phase (19). Morroniside was found to restore the H_2_O_2_-induced imbalance of the S phase only but not staurosporine-induced G2 phase imbalance, suggesting that morroniside may be an S phase-specific regulator.

Results of the present study imply that the downregulation of Rb protein expression may be relevant to S phase regulation. It is well known that Rb protein plays roles throughout the cell cycle. The phosphorylation and dephosphorylation of Rb play key roles in the cell cycle (20). Usually, Rb protein levels undergo no significant change through the cell cycle (21). However, results of the present study showed that Rb protein was markedly downregulated by morroniside. Morronisde also caused the rebalance of the S phase length in HELF cells, which was previously increased by H_2_O_2_ treatment. It remains unclear how Rb protein levels are involved in S phase regulation. Identification of the signaling pathways regulated by morroniside and the molecular mechanisms regulating Rb protein stability require further *in vitro* and *in vivo* study.

In conclusion, morroniside was found to inhibit apoptosis in normal HELF cells but not in the A549 cancer cell line. These effects included the protection of cell proliferation and normal cell morphology, and the restoration of the S phase to normal levels. Furthermore, the interaction between morroniside and H_2_O_2_ signaling was found to be involved in HELF proliferation and apoptosis. Thus, morroniside is a potential compound for clinical amelioration of the side effects of anticancer treatments.
